# Overlooked signs and symptoms: Why leprosy is often misdiagnosed

**DOI:** 10.1016/j.jdcr.2026.01.055

**Published:** 2026-02-09

**Authors:** Tong Ba Tran, Thuy Thi Phan Nguyen, Ngoc Thi Kim Pham, Quan Duy Nguyen, Doan Minh Phan, Chau Thuy Ai Nguyen, Dam Van Pham, Nhi Thi Uyen Pham

**Affiliations:** Ho Chi Minh City Hospital of Dermato-Venereology, Ho Chi Minh City, Vietnam

**Keywords:** AFB, atypical presentation, erythema lesions, fungal infection, Hansen’s disease, hypopigmented lesions, leprosy, lupus erythematosus, mycobacterium

## Introduction

Leprosy (Hansen’s disease) remains a relevant public health concern in countries like Vietnam, where new cases continue to be reported annually despite global efforts to reduce its prevalence. According to the Vietnam National Leprosy Control Program, dozens of new cases are still diagnosed each year, particularly in areas with limited health care access. Leprosy is caused by “*Mycobacterium leprae*” and classically presents with hypopigmented or erythematous skin lesions, peripheral nerve thickening, and sensory loss. However, early or atypical forms may lack these features, leading to misdiagnosis.

The disease can mimic various dermatologic and systemic conditions, such as lupus erythematosus, Sweet’s syndrome, fungal infections, erythema nodosum necroticans, or mycosis fungoides.^1^ In nonspecialized settings, this clinical overlap often results in prolonged, ineffective treatment before the correct diagnosis is established.^1^^,^^2^ At our dermatology hospital, we encountered several such cases.

In this series, we report on 5 patients who were initially misdiagnosed and treated for conditions—such as superficial fungal infection, cutaneous lupus erythematosus, and hemangioma—before histopathology confirmed leprosy. These cases highlight the protean nature of the disease and underscore the importance of considering leprosy in the differential diagnosis of chronic, treatment-resistant dermatoses. Early recognition is essential to avoid complications and prevent transmission.

## Case series

### Case 1

**Fig 1:**
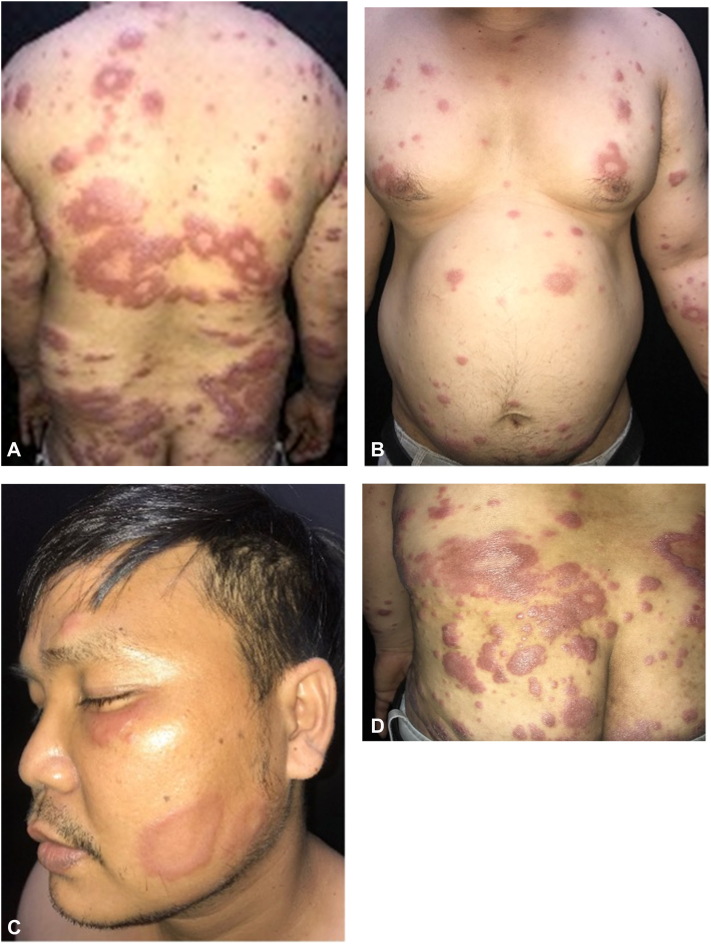
**A-D,** Multiple annular erythematous plaques on the face, trunk, and extremities.

A 37-year-old male presented with a 1-year history of progressive, asymptomatic, annular erythematous plaques involving the face, trunk, and extremities (Fig 1, *A-D*). Over this period, he consulted multiple dermatologists, all of whom diagnosed a superficial fungal infection and prescribed topical and systemic antifungal agents, including terbinafine and itraconazole (2 weeks to 1 month per course). Repeated potassium hydroxide preparations were negative, which was attributed to prior use of antifungals. Due to fragmented follow-up across different providers, the lack of therapeutic response was not initially recognized.

Cutaneous examination revealed multiple annular, erythematous plaques and papules with a shiny surface distributed on the face, trunk, and extremities. A subset of the plaques exhibited a characteristic “targetoid” morphology, with a sharply demarcated inner border and an indistinct, gradually fading outer rim. Neurological assessment showed no thickening of the ulnar, tibial, or facial nerves, and sensory function within the lesions was preserved. Given the chronicity and poor response to antifungals, the differential diagnosis included mycosis fungoides and lepromatous leprosy.

An incisional biopsy of a representative lesion demonstrated foamy macrophages within the dermis. Fite staining revealed abundant acid-fast bacilli (AFB) and globi, confirming the diagnosis of lepromatous leprosy.

### Case 2

**Fig 2:**
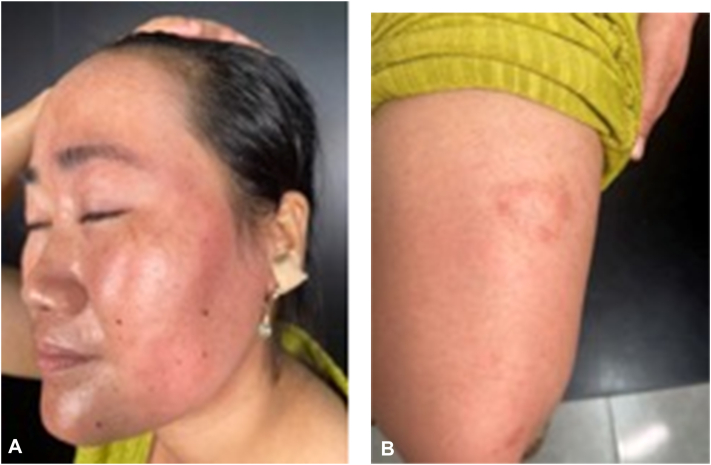
**A** and **B,** Annular erythematous plaques on the face and the left lower extremity.

A 35-year-old woman presented with a 1-year history of persistent, nonpruritic erythematous plaques. The lesions first appeared as bright red annular patches on the lower legs, gradually spreading to the arms, thighs, and left side of the face (Fig 2, *A* and *B*). She had been evaluated at multiple clinics and diagnosed with dermatophytosis, for which she received various topical and systemic antifungal treatments with slight improvement. No skin scraping potassium hydroxide preparation was performed prior to this consultation. The presumptive diagnosis of dermatophytosis (Tinea faciei) was based primarily on the annular configuration of the erythematous plaques.

On examination, the plaques were well-demarcated, erythematous, and annular in some areas, distributed on the extremities and left hemiface. Hypoesthesia and paresthesia were noted over several plaques, although protective sensation was preserved. No motor deficits were found on neurological assessment. Of note, her husband had been receiving treatment for leprosy for the past 2 years.

A skin biopsy from a lesion on the leg revealed mild epidermal atrophy and a diffuse inflammatory infiltrate in the dermis, composed of lymphocytes, histiocytes, and scattered epithelioid cells. Some infiltrates formed granulomas around adnexal structures. Ziehl–Neelsen staining demonstrated the presence of acid-fast bacilli (AFB). These histopathological features were consistent with borderline tuberculoid (BT) leprosy. However, the slit-skin smear taken from the earlobe was negative for AFB.

A final diagnosis of BT leprosy was made, and the patient was started on multidrug therapy as per World Health Organization guidelines.

### Case 3

**Fig 3:**
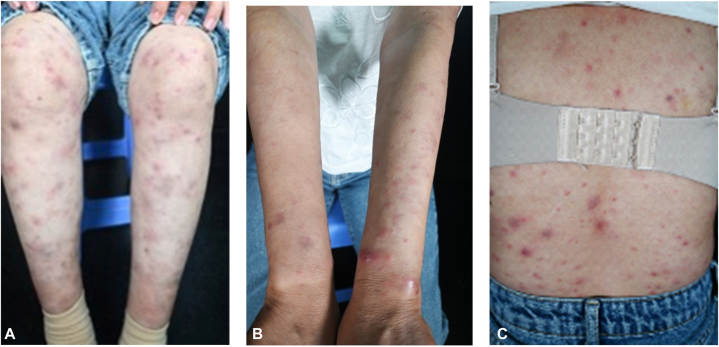
**A-C,** Multiple erythematous plaques and nodules on the trunk and extremities.

A 39-year-old female presented with a 3-year history of recurrent, painful, erythematous papules, plaques, and nodules on her trunk and extremities (Fig 3, *A*-*C*). These episodes were accompanied by fever and loss of appetite. She had previously been diagnosed with cutaneous lupus and treated with systemic methylprednisolone; however, her condition rapidly relapsed upon tapering the dosage. Critically, no diagnostic skin biopsy had been performed prior to presentation. Serological tests for lupus, including antinuclear antibody and anti-dsDNA, were negative. In the absence of definitive histopathological confirmation, the initial lupus diagnosis was made based solely on the clinical presentation and serological findings. Nevertheless, this serological profile, coupled with the severe and long-standing clinical symptoms, made a diagnosis of cutaneous lupus less likely.

**Fig 4:**
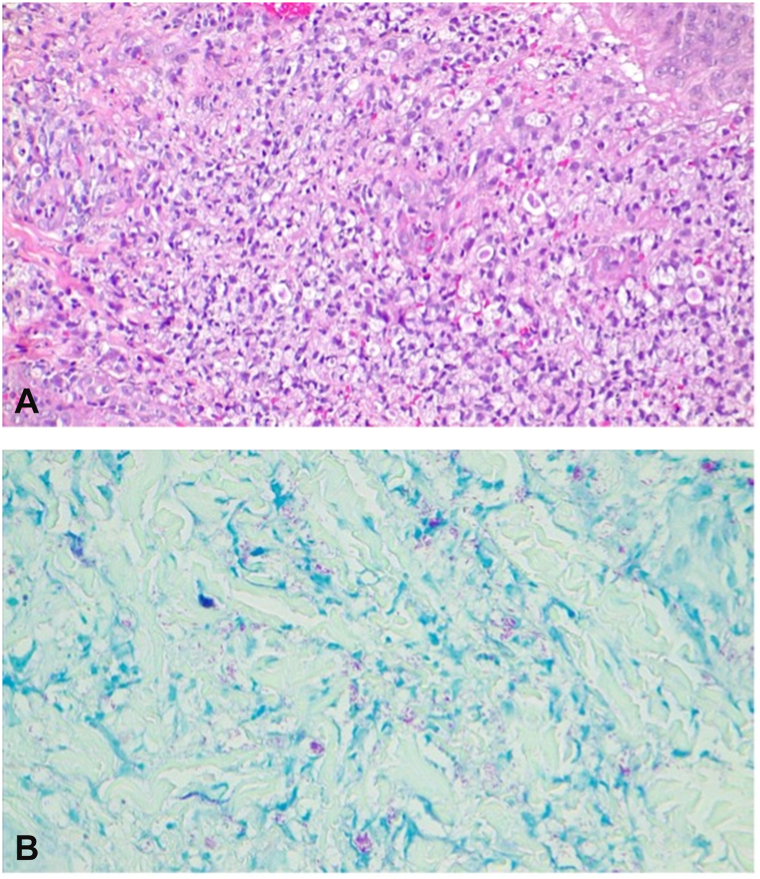
**A** and **B,** Diffuse infiltration of foamy cells in the dermis with positive AFB. *AFB*, Acid-fast bacilli.

Given the clinical presentation of recurrent erythematous nodules accompanied by fever and loss of appetite, other differential diagnoses were considered, including erythema nodosum and Sweet's syndrome. A skin biopsy was recommended. Histological examination revealed aggregates of foamy cells in the superficial dermis and around adnexal structures. No evidence of leukocytoclastic vasulitis was identified. Fite staining was positive. The final diagnosis was lepromatous leprosy with a type 2 reaction (erythema nodosum leprosum) (Fig 4, *A* and *B*). The patient was initiated on multidrug therapy, leading to a gradual resolution of the lesions over time.

### Case 4

**Fig 5:**
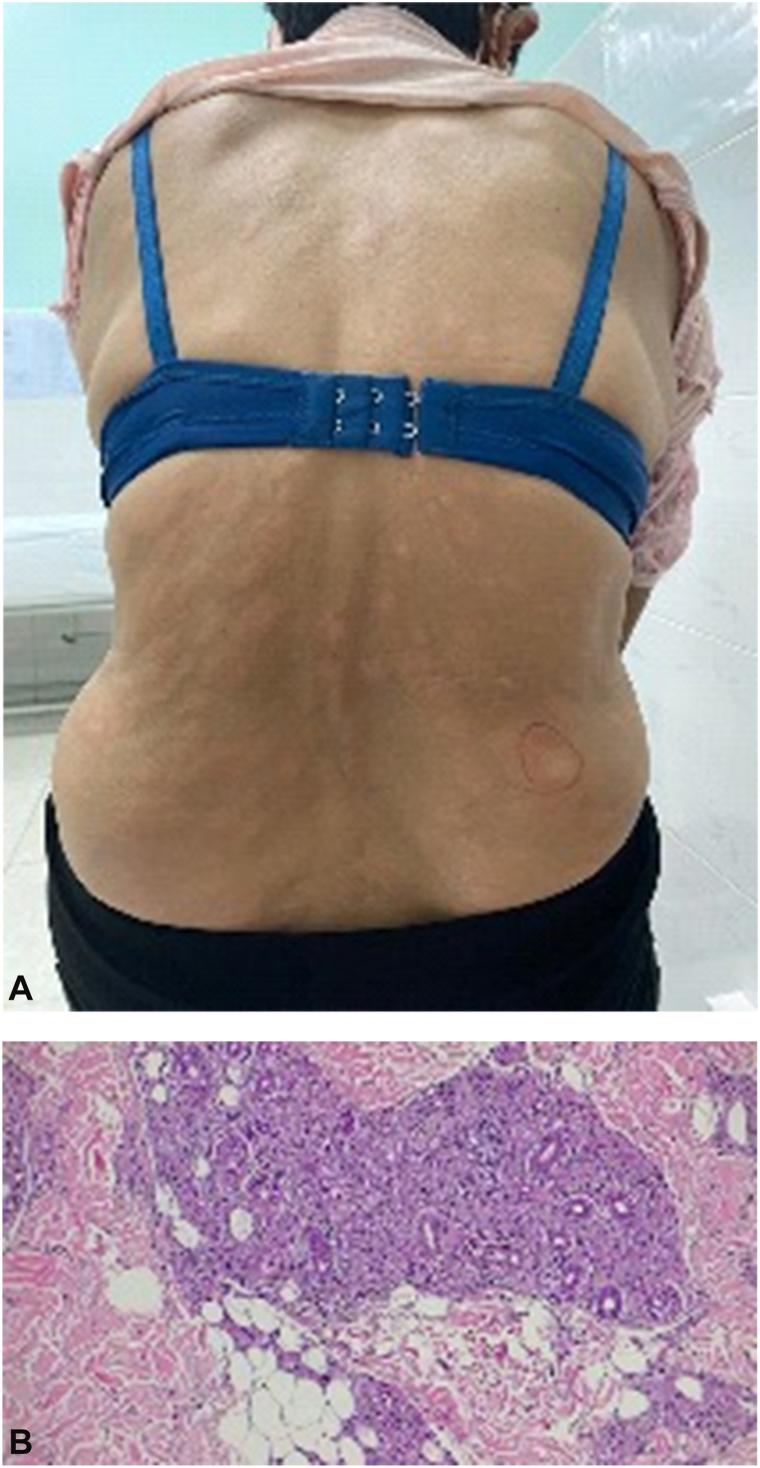
**A,** Asymptomatic erythematous plaques, pale pink in color, with relatively well-demarcated borders and symmetrically distributed on both sides of the trunk. **B,** Poorly defined granuloma formed by dense inflammatory infiltration composed of lymphocytes, histiocytes, and scattered epithelioid cells.

A 60-year-old female presented with a 2-year history of gradually progressive, asymptomatic erythematous plaques on the back, chest, arms, and face. The lesions were round to oval in shape, measuring 1-3 cm in diameter, and pale pink in color, with relatively well-demarcated borders, symmetrically distributed on both sides of the trunk (Fig 5, *A*). Laboratory investigations revealed a positive antinuclear antibody test, while other routine blood tests were within normal limits. No skin biopsy was performed at that time. Based on the clinical presentation, a diagnosis of cutaneous lupus erythematosus was made, and the patient was treated with hydroxychloroquine 200 mg/day and topical corticosteroids for over 1 year, with minimal clinical improvement.

Subsequently, a skin biopsy was performed, showing mild epidermal atrophy. In the superficial and mid-dermis, a moderately dense inflammatory infiltrate composed of lymphocytes, histiocytes, and scattered epithelioid cells was observed. These infiltrates formed poorly defined granulomas involving neurovascular bundles and adnexal structures (Fig 5, *B*). Perineural lymphocytic infiltration was also noted. Ziehl–Neelsen staining demonstrated numerous AFB, present singly and in clusters. These histopathological features were consistent with a diagnosis of borderline-borderline leprosy. Bacterial index testing revealed a value of 4+ at 3 different anatomical sites.

Based on these findings, the final diagnosis of borderline-borderline leprosy was established, and the patient was commenced on multidrug therapy in accordance with World Health Organization guidelines.

### Case 5

**Fig 6:**
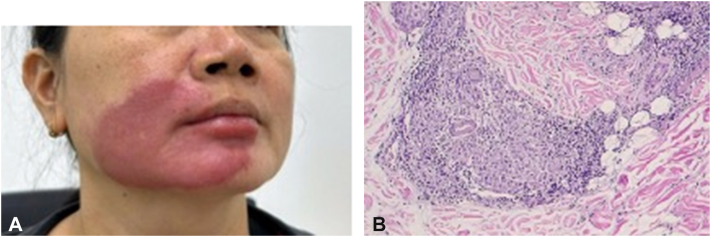
**A,** Well-demarcated, erythematous plaque involving the right cheek and perioral area. **B,** Dermal infiltration by lymphocytes, histiocytes, and scattered giant cells.

A 48-year-old female patient presented with a 6-month history of an annular erythematous plaque on the right perioral facial region (Fig 6, *A*). The lesion was nonpruritic, painless, and had not increased in size over time. During this period, she had self-treated with unspecified topical agents at home without improvement and had not sought formal medical evaluation or undergone any diagnostic testing.

Dermatological examination revealed a well-demarcated dark-red erythematous plaque, 10 cm in diameter, located on the right perioral facial area. No similar lesions were noted elsewhere. Sensory function in the affected area was preserved. Due to the chronic course and lesion location, differential diagnoses included hemangioma, lupus vulgaris, and lupus erythematosus tumidus.

A biopsy sample (0.6 × 0.3 cm) from the right cheek showed epidermal hyperplasia with acanthosis and hyperkeratosis. The dermis exhibited infiltration by inflammatory cells, including lymphocytes, histiocytes, and scattered epithelioid cells. Granulomatous formation with neovascularization and fibrosis was observed in some areas (Fig 6, *B*). Ziehl–Neelsen staining did not reveal any AFB, suggesting an inflammatory granuloma consistent with BT leprosy.

## Discussion

The first 2 cases emphasize the diagnostic challenges associated with chronic annular plaques. When classical signs, such as marked sensory loss or nerve thickening, are subtle or absent, leprosy may be mistaken for more common dermatoses, like dermatophytosis. Several reports have documented similar diagnostic pitfalls when leprosy was misdiagnosed as a tinea infection and treated unsuccessfully with antifungals before histopathology confirmed the diagnosis.^2^^,^^3^ Lack of response to antifungal therapy and repeated negative skin scraping should always prompt an alternative diagnosis and histopathological examination. Also, history of close household contact should have raised early suspicion of leprosy, as in the third case. The first case also highlights the importance of consistent clinical follow-up by the same provider whenever feasible. When continuity of care is not possible, serial photographic documentation by either the clinician or the patient can aid in assessing treatment response and alert the follow-up physician when the patient failed to respond to previous treatment.

The next 2 cases highlight other mimickers that leprosy could be mistaken for, that is, Sweet’s syndrome and cutaneous lupus. Cases of erythema nodosum leprosum mimicking Sweet's syndrome have been reported in the medical literature, indicating a degree of clinical overlap between these conditions. Besides their similar skin manifestations, the diagnostic challenge is heightened because both conditions can lead to leukocytosis and demonstrate a rapid response to oral corticosteroids, making differentiation difficult.^1^^,^^4^

Regarding lupus, in the third case, although it can manifest with various lesions, including subcutaneous nodules in lupus panniculitis and erythematous plaques and papules in subacute lupus, the negative serology in this patient, especially given the severity and chronicity of her symptoms, made a lupus diagnosis less probable. In the fourth case, the diagnosis was more challenging with a positive antinuclear antibody and symmetrical erythematous plaques with well-defined borders, distributed over both photo-exposed and photo-protected areas. Several reports have documented similar diagnostic dilemmas.^5^

These cases highlight how overlapping clinical features—including lesion morphology, distribution, and serological findings—can obscure the correct diagnosis. What differentiates the current case is the chronicity of the lesions and their resistance to standard lupus therapy, prompting further investigation. The presence of perineural lymphocytic infiltration and the identification of AFB on Ziehl–Neelsen staining were key diagnostic clues indicating leprosy. These findings are pathognomonic and underscore the importance of histopathological evaluation in cases with atypical or refractory cutaneous manifestations.^5^, ^6^, ^7^

The final case illustrates an additional misdiagnosis of BT as lupus vulgaris, another infectious granulomatous disease that may present with overlapping clinical features, particularly when involving facial lesions.^8^ BT leprosy typically presents with clinical features suggestive of diagnosis, such as annular plaques with satellite lesions (often more than 5), asymmetrical distribution, sensory loss in the lesions, and less sharply defined borders compared to tuberculoid leprosy. In our patient, the clinical presentation led to a classification of BT, rather than pure tuberculoid leprosy (TT). This distinction was based on 2 key features. First, the lesion was unusually extensive, measuring over 10 centimeters in its greatest diameter, which is uncommon for classic TT. Second, the lesion's border was diffuse and blurred on the right side, contrasting with the sharply demarcated lesions typically seen in TT. Our patient also exhibited atypical signs of BT leprosy that could be mistaken for lupus vulgaris like the absence of documented sensory loss, leading to delayed diagnosis and suboptimal treatment.

## Conclusion

This case series highlights the diverse and often misleading presentations of leprosy, which can mimic various chronic dermatoses. Delayed diagnosis may result from subtle or absent classical signs and failure to consider leprosy in treatment-resistant cases. Clinicians should maintain a high index of suspicion when evaluating persistent skin lesions. Early biopsy and histopathological confirmation are crucial for preventing misdiagnosis and avoiding long-term complications. Timely recognition ensures appropriate treatment and limits the progression of the disease.

## Conflicts of interest

None disclosed.

## References

[1] Aires N.B., Refkalefsky Loureiro W., Villela M.A.C., Sakai Valente N.Y., Trindade M.Â.B. (2009). Sweet's syndrome type leprosy reaction. J Eur Acad Dermatol Venereol.

[2] Alfaragi M., Ahmed F., Osman W. (2022). Challenges related to the cases of lepromatous leprosy: a report of two cases. Pan Afr Med J.

[3] Daroach M., Bhallavi H., Narang T. (2022). Leprosy masquerading as tinea faciale. Ann Natl Acad Med Sci (India).

[4] Chiaratti F.C., Daxbacher E.L.R., Neumann A.B.F., Jeunon T. (2016). Type 2 leprosy reaction with Sweet's syndrome-like presentation. An Bras Dermatol.

[5] Youssef H., Mahani T., Hojjati M. (2023). Leprosy, the great imitator of rheumatic diseases: a case study. Cureus.

[6] Ridéey D.S., Jopling W.H. (1966). Classification of leprosy according to immunity. A five-group system. Int J Lepr Other Mycobact Dis.

[7] Yadav V., Verma D., Mendiratta V. (2024). Uncustomary and rare presentation of Hansen’s disease ‘a mimicker’-a narrative review. Lepr Rev.

[8] Yu-Ying L., Jian L., Hui C., Hsuan-Wei C., Wei Z. (2016). Two cases of leprosy misdiagnosed in a family, one elephantiasis-like with lepromatous leprosy. Lepr Rev.

